# GPR120: A bi-potential mediator to modulate the osteogenic and adipogenic differentiation of BMMSCs

**DOI:** 10.1038/srep14080

**Published:** 2015-09-14

**Authors:** Bo Gao, Qiang Huang, Qiang Jie, Wei-Guang Lu, Long Wang, Xiao-Jie Li, Zhen Sun, Ya-Qian Hu, Li Chen, Bao-Hua Liu, Jian Liu, Liu Yang, Zhuo-Jing Luo

**Affiliations:** 1 Institute of Orthopedic Surgery, Xijing Hospital, Fourth Military Medical University, Xi’an 710032, People’s Republic of China; 2 Lanzhou General Hospital of Lanzhou Military Command, Lanzhou Gansu, 730050, People’s Republic of China; 3 KMEB, Molecular Endocrinology, Campusvej 55, DK-5230 Odense M, Denmark; 4 Health Science Center, Shenzhen University, 3688 Nanhai Ave, Shenzhen 518060, People’s Republic of China

## Abstract

Free fatty acids display diverse effects as signalling molecules through GPCRs in addition to their involvement in cellular metabolism. GPR120, a G protein-coupled receptor for long-chain unsaturated fatty acids, has been reported to mediate adipogenesis in lipid metabolism. However, whether GPR120 also mediates osteogenesis and regulates BMMSCs remain unclear. In this study, we showed that GPR120 targeted the bi-potential differentiation of BMMSCs in a ligand dose-dependent manner. High concentrations of TUG-891 (a highly selective agonist of GPR120) promoted osteogenesis via the Ras-ERK1/2 cascade, while low concentrations elevated P38 and increased adipogenesis. The fine molecular regulation of GPR120 was implemented by up-regulating different integrin subunits (α1, α2 and β1; α5 and β3). The administration of high doses of TUG-891 rescued oestrogen-deficient bone loss *in vivo*, further supporting an essential role of GPR120 in bone metabolism. Our findings, for the first time, showed that GPR120-mediated cellular signalling determines the bi-potential differentiation of BMMSCs in a dose-dependent manner. Additionally, the induction of different integrin subunits was involved in the cytoplasmic regulation of a seesaw-like balance between ERK and p38 phosphorylation. These findings provide new hope for developing novel remedies to treat osteoporosis by adjusting the GPR120-mediated differentiation balance of BMMSCs.

Osteoporosis (OP) is characterized by a decrease in bone mass and density accompanied by a dramatic change in the osteoblast/adipocyte ratio in the bone marrow cavity^1^,^2^. The abnormal osteoblast/adipocyte ratio is determined by the imbalance between the decreased osteogenic and increased adipogenic differentiation tendency of bone marrow mesenchymal stem cells (BMMSCs)^3^, and this imbalance will cause more fat to reside in the bone marrow cavity, which in turn modulates the bone formation process, ultimately causing osteoporosis^4^. Many core factors, such as β-catenin, Runx2 and Ppar-γ, are highly involved in the bi-potential differentiation process of BMMSCs^5^,^6^,^7^; however, little evidence has shown how BMMSCs sense the extracellular signals and change their differentiation tendency in the first place.

Free fatty acids (FFAs) display diverse effects as signalling molecules through a few G protein-coupled receptors (GPCRs) in addition to their involvement in cellular metabolism. Ligands bind specifically to GPCRs to stimulate and induce various cellular responses via several second messenger pathways—e.g., modulation of cAMP production, the phospholipase C pathway, Ca^2+^ channels, and mitogen-activated protein kinases^8^,^9^,^10^.

In recent decades, GPR40 and GPR120, which have been shown to be activated by FFAs, were reported to be expressed in bone-related cells^11^,^12^,^13^. Wauquier *et al*. and Gao *et al*. reported that GPR40 was expressed in osteoclasts, osteocytes and BMMSCs and protected bone loss via the inhibition of osteoclast differentiation and mediated oestrogen-induced osteogenesis of BMMSCs^11^,^14^,^15^.

GPR120, a G protein-coupled receptor for long-chain unsaturated fatty acids, has been reported to mediate adipogenesis and glucagon-like peptide-1 secretion in lipid metabolism^16^. GPR120 was also found to be involved in the differentiation regulation of macrophagy and chondrocytes^17^,^18^. Moreover, GPR120 was the specific membrane receptor of ω-3 PUFA (DHA and EPA), which was reported to accelerate osteoblast differentiation and proliferation^19^,^20^,^21^,^22^. Additionally, in our recent study, we showed that GPR120 mediated Ginsenoside-Rb2-induced proliferation and the anti-apoptosis effects of BMMSCs^23^, also suggesting a possible role of GPR120 in bone metabolism. However, whether GPR120 directly senses extracellular signals and mediates osteoblast differentiation and the fine regulation of BMMSCs by FFAs remain unclear.

Tsujimoto and San-Gun Roh’s study showed that GPR120 mediated the differentiation and maturation of adipocytes both *in vivo* and *in vitro*^24^,^25^. Therefore, we speculated that GPR120 might be the “button” to modulate osteogenesis as well as GPR120-mediated adipogenesis of BMMSCs, and these two events might be underway through different molecular mechanisms.

Our results, for the first time, demonstrated that, *in vitro*, ligand dosage-dependent GPR120 is a bi-potential mediator to modulate the osteogenic and adipogenic differentiation process of BMMSCs by activating different integrin families, such as integrin α1β1, integrin α2β1 and integrin αvβ3. *In vivo*, we found that the injection of the agonist of GPR120 into the bone marrow cavity significantly reversed the process of bone loss. These findings indicated that GPR120, currently a target for the treatment of type 2 diabetes^26^,^27^,^28^, and the newly found “obesity gene” that forecasts obesity, could have bonus effects on the prevention of osteoporosis and obesity in the future.

## Results

### GPR120 positively regulates the osteogenesis of BMMSCs

**Figure 1:**
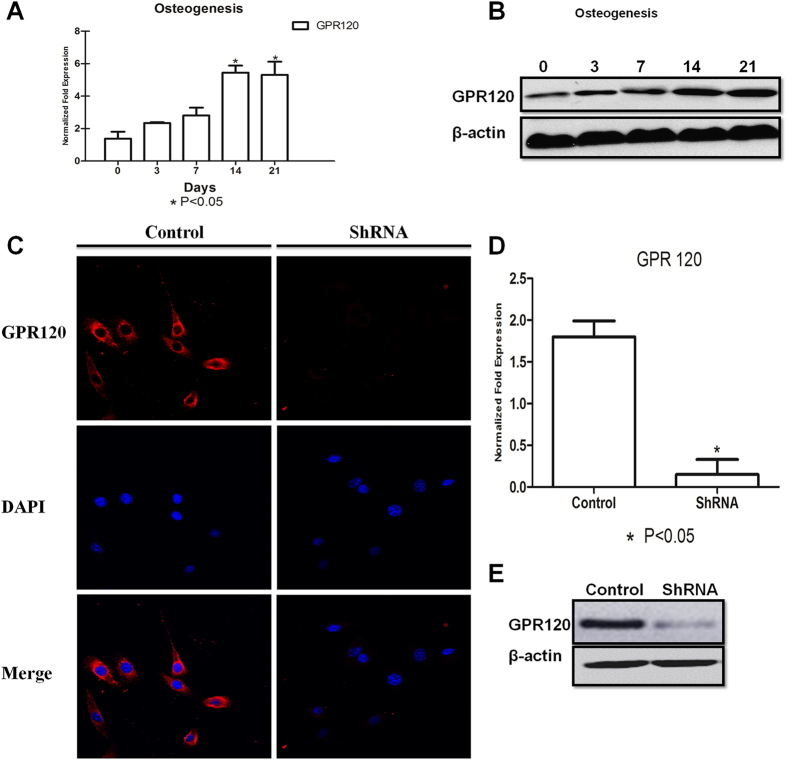
GPR120 positively regulates the osteogenesis of BMMSCs. BMMSCs were cultured for 0, 3, 7, 14 and 21 days of osteogenesis, respectively. (**A**) RT-PCR and (**B**) western blot analysis of GPR120. BMMSCs were invalidated for GPR120 expression or transfected with a nontargeting vector. (**C**) Immunocytochemistry shows the expression of GPR120 (red) by BMMSCs. The merged image shows GPR120 with DAPI nuclear staining. (**D**) RT-PCR and (**E**) western blot analysis of GPR120. The expression of each target gene was calculated as the relative expression to β-actin and is represented as normalized fold expression. Data are represented as the mean ± SD of 3 independent experiments. *P < 0.05 and **P < 0.01 compared with the control group.

First, we detected the expression of GPR120 transcripts in primary BMMSCs and found that the expression level of GPR120 was gradually increased during osteogenic induction. Moreover, the level of GPR120 protein after 14 and 21 days of induction was much higher than that after 3 and 7 days of induction (Fig. 1A,B), suggesting a possible role of GPR120 in the osteoblast differentiation of BMMSCs.

**Figure 2:**
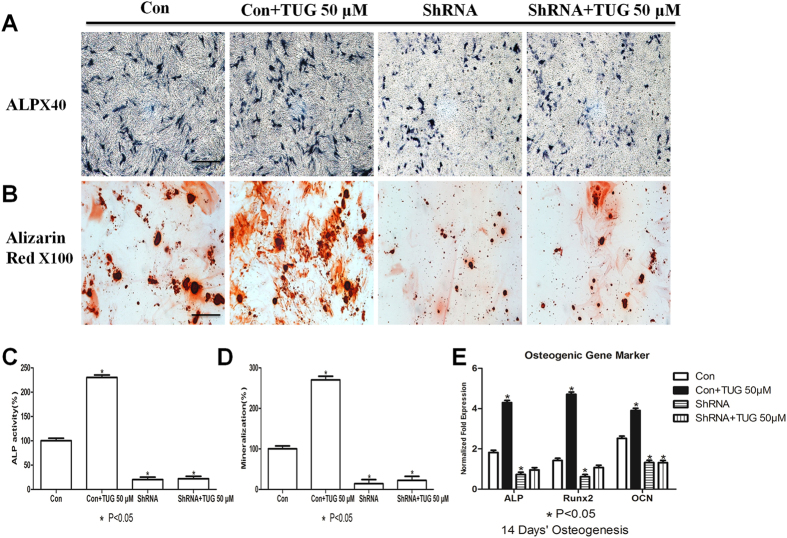
Lack of GPR120 negatively impacts the osteogenesis of BMMSCs. Control and GPR120 shRNA BMMSCs were incubated for 14 days of osteogenesis in the presence or absence of 50 μM TUG-891. (**A**) ALP (scale bar = 200 μm) and (**B**) Alizarin red (scale bar = 80 μm) staining of cells. (**C**) Difference in the ALP activity of osteogenic differentiation. The control value for ALP activity was 0.415 ± 0.020 units/mg protein. (**D**) Difference in the mineralization of osteogenic differentiation. The control value for mineralization was 0.910 ± 0.013 OD. (**E**) Difference in the osteogenic mRNA expression of *Alp, Runx2 and Ocn*. The expression of each target gene was calculated as the relative expression to β-actin and is represented as normalized fold expression. Data are represented as the mean ± SD of 3 independent experiments. *P < 0.05 and **P < 0.01 compared with the control group.

To further explore the role of GPR120 in osteogenesis, GPR120 was knocked down in BMMSCs using stable shRNA interference. Immunostaining, western blotting and RT-PCR showed that the expression of GPR120 was significantly reduced to an extremely low level (Fig. 1C–E). The potential of osteoblast differentiation of these BMMSCs was evaluated by ALP and Alizarin Red staining in the presence or absence of 50 μM TUG-891, which is a highly selective agonist of GPR120^29^. Statistical analyses of both ALP and Alizarin Red staining showed that the knock-down of GPR120 weakened the osteogenesis of BMMSCs dramatically; however, in the presence of 50 μM TUG-891, the ability of osteogenesis was not altered (Fig. 2A–D). These results showed that GPR120 mediated the osteogenic differentiation of BMMSCs, at least partially. These data were further confirmed by comparing the expression of osteogenic markers. *Alp*, *Runx2* and *Ocn* were significantly down-regulated when GPR120 was knocked down, and TUG-891 did not rescue this effect (Fig. 2E).

### GPR120 promotes the osteogenesis of BMMSCs via the Ras-ERK1/2 signalling pathway

**Figure 3:**
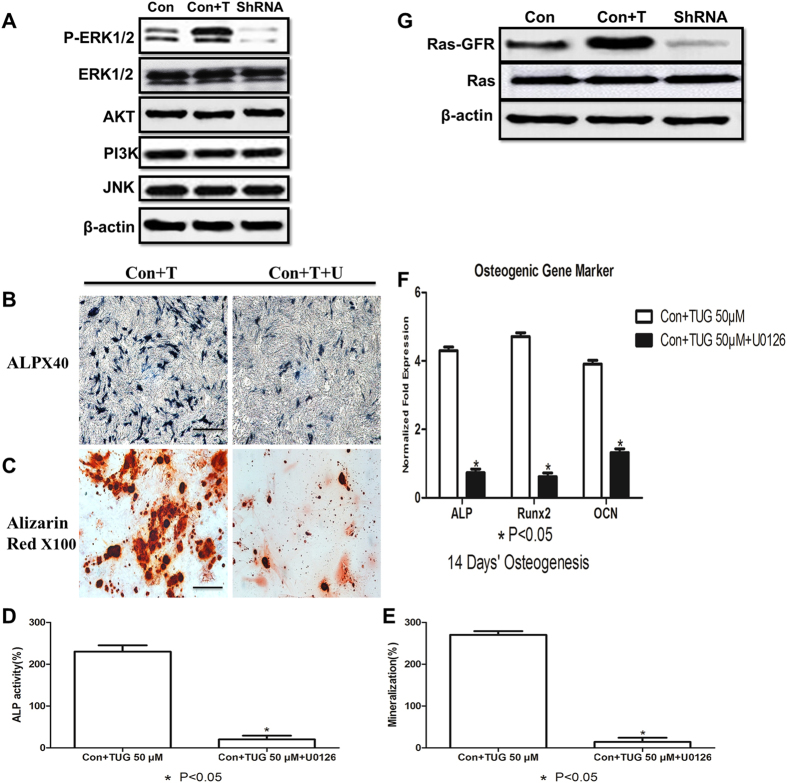
GPR120 promotes the osteogenesis of BMMSCs via the Ras-Erk1/2 cascade. (**A**) Western blotting analysis of ERK1/2, pERK1/2, Akt, PI3K and JNK. Con: Control cells, Con + T: Control cells incubated with 50 μM TUG-891, shRNA: GPR120 shRNA cells (**B**) ALP (scale bar = 200 μm) and (**C**) Alizarin red staining (scale bar = 80 μm) of cells. (**D**) ALP activity of osteogenic differentiation between Con + T and Con + T + U BMMSCs. The control value for ALP activity was 0.720 ± 0.015 units/mg protein. (**E**) Mineralization of the osteogenic differentiation between Con + T and Con + T + U BMMSCs. The control value for mineralization was 0.985 ± 0.012 OD. Con + T: Control cells incubated with 50 μM TUG-891, Con + T + U: Control cells incubated with 50 μM TUG-891 plus10 μM U0126. (**F**) Osteogenic mRNA expression of *Alp, Runx2 and Ocn*. (**G**) Western blotting analysis of Ras and Ras-GRF. The expression of each target gene was calculated as the relative expression to β-actin and is represented as the normalized fold expression. Data are represented as the mean ± SD of 3 independent experiments. *P < 0.05 and **P < 0.01 compared with the Con + T group.

To elucidate the underlying mechanism of GPR120 in osteoblast differentiation, the expression level of phosphate ERK1/2 (pERK1/2) and total ERK1/2, AKT, PI3K and JNK were compared in the osteogenesis-induced primary BMMSCs. The western blotting results showed that pERK1/2 was obviously increased when GPR120 was activated by TUG-891 and was significantly reduced when GPR120 was knocked down. However, the expression levels of total ERK1/2, AKT, PI3K and JNK were not significantly changed by the stimulus (Fig. 3A).

To further confirm that the phosphorylation and activation of the ERK1/2 pathway were essential for the GPR120-mediated osteoblast differentiation of BMMSCs, U0126, an inhibitor of the ERK1/2 pathway, was used. Primary BMMSCs were cultured in osteogenic medium in the presence of 50 μM TUG-891 and/or 10 μM U0126. The results of ALP and Alizarin Red staining showed that U0126 could significantly block the osteogenic induction effect of TUG-891 (Fig. 3B–E). Moreover, the expression of osteogenic markers *Alp*, *Runx2* and *Ocn* was significantly reduced in the presence of U0126 (Fig. 3F). To further uncover the upstream regulator of ERK1/2 in the osteogenic induction of BMMSCs, we tested the Ras kinase activity. Figure 3G showed that the activation of GPR120 elevated the expression level of Ras-GRF, which is the active form of Ras, while the knockdown of GPR120 dramatically reduced the levels of Ras-GRF, further indicating that GPR120 might promote the osteogenic differentiation of BMMSCs via the Ras-ERK1/2 signalling pathway.

### The GPR120 effects on osteogenesis are exerted in a ligand dose-dependent manner

Tsujimoto and colleagues showed that GPR120-deficient mice that were fed a high-fat diet developed obesity, glucose intolerance and fatty liver with decreased adipocyte differentiation and lipogenesis^25^. This discrepancy of GPR120 in adipocyte differentiation and osteoblast differentiation led us to further study the bi-potential differentiation of BMMSCs with the activation of GPR120 *in vitro* and *in vivo*.

**Figure 4:**
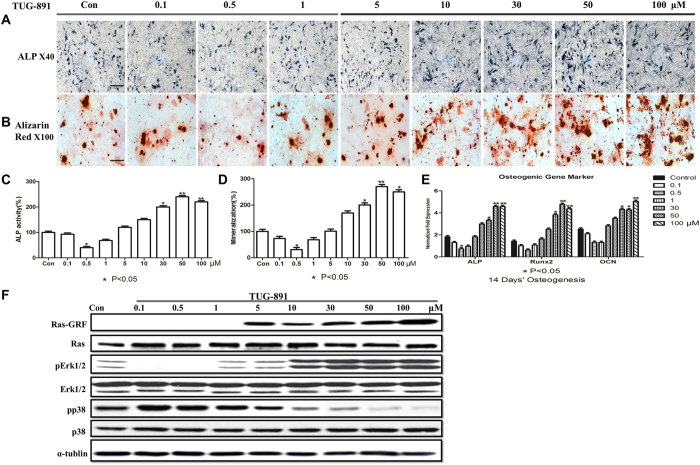
TUG-891, an agonist of GPR120, significantly promotes the osteogenesis of BMMSCs at high concentrations. BMMSCs were incubated for 14 days of osteogenesis in the presence of different concentrations of TUG-891 [0 (Con), 0.1, 0.5, 1, 5, 10, 30, 50, 100 μM]. (**A**) ALP (scale bar = 200 μm) and (**B**) Alizarin red (scale bar = 80 μm) staining of cells. (**C**) Effect of TUG-891 on the ALP activity of the osteogenic differentiation of BMMSCs. The control value for ALP activity was 0.432 ± 0.015 units/mg protein. (**D**) Effect of TUG-891 on the mineralization of the osteogenic differentiation of BMMSCs. The control value for mineralization was 0.920 ± 0.016 OD. (**E**) Effect of TUG-891 on the osteogenic mRNA expression of *Alp, Runx2 and Ocn*. (**F**) Western blot analysis of ERK1/2, pErk1/2, Ras, Ras-GRF, p38 and pp38. The expression of each target gene was calculated as the relative expression to β-actin and is represented as normalized fold expression. Data are represented as the mean ± SD of 3 independent experiments. *P < 0.05 and **P < 0.01 compared with the control group.

TUG-891 is a highly selective agonist of GPR120 compared with other agonists^30^. We added TUG-891 in increasing concentrations from 0 to 100 μM (0, 0.1, 0.5, 1, 5, 10, 30, 50, 100 μM) to the osteogenic medium for 14 days. The results showed that high concentrations of TUG-891 (30, 50, 100 μM) markedly increased ALP staining and the mineralized matrix area compared with low concentrations of TUG-891 (0.1, 0.5, 1 μM) and control cells (P < 0.05, Fig. 4A–D). High concentrations of TUG-891 (30, 50, 100 μM) also significantly improved the expression level of osteogenic gene markers, while there were no significant changes in cells cultured with low concentrations (0.1, 1, 5 μM) (P < 0.05, Fig. 4E). Additionally, 0.5 μM TUG-891 significantly weakened the osteogenic ability of BMMSCs, indicating that a low concentration of TUG-891 might have a reverse effect to interfere with the osteogenic process. Furthermore, 0.5 μM TUG-891 reduced the expression of *Alp* but not that of *Runx2* and *Ocn* (P < 0.05, Fig. 4E).

The western blotting results showed that high concentrations of TUG-891 (30, 50 and 100 μM) markedly activated Ras-GRF and ERK1/2 phosphorylation, while total Ras and ERK1/2 did not obviously change (Fig. 4F). Low concentrations of TUG-891 (0.1, 0.5 and 1 μM) were found to not activate the Ras pathway but downregulated the phosphorylation of ERK1/2, suggesting that there might be another molecule participating in the inhibition of pERK1/2 in the presence of low concentrations of TUG-891 (Fig. 4F).

A study by Olsen and Volloch indicated that p38 could repress the phosphorylation of ERK and subsequently promote adipogenesis while inhibiting osteogenesis^31^. Therefore, p38, a key negative regulator of ERK, was detected during the osteoblast differentiation of BMMSCs with a sequential concentration of TUG-891. We found that the phosphorylation of p38 was up-regulated in the presence of low concentrations of TUG-891 (0.1, 0.5 μM). However, p38 phosphorylation was down-regulated in the presence of high TUG-891 concentrations (Fig. 4F), indicating that a low concentration of TUG891, an agonist of GPR120, might inhibit osteoblast differentiation through p38-mediated inhibition of the ERK pathway.

### Low concentrations of TUG-891 play a positive role in inducing the adipogenesis of BMMSCs

**Figure 5:**
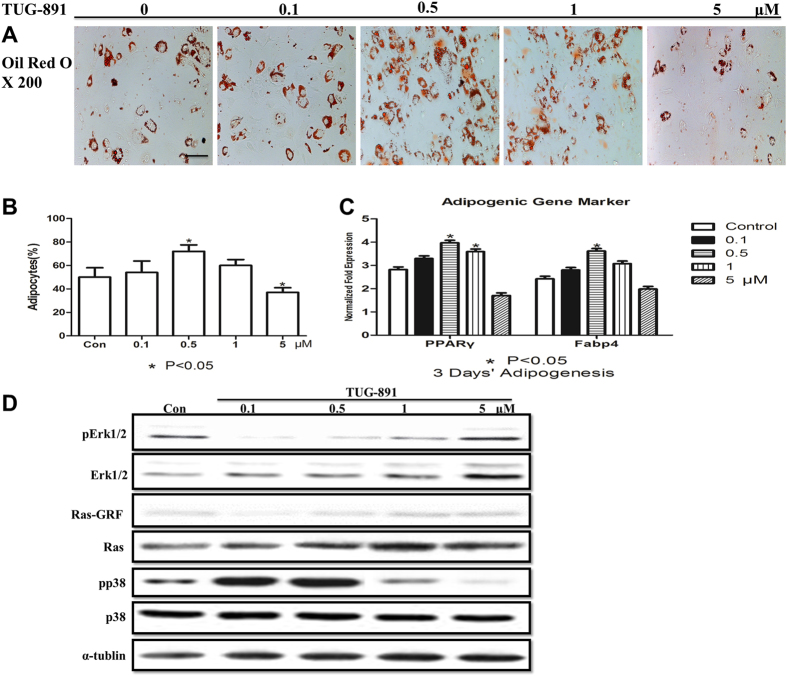
Low concentrations of TUG-891 induce the adipogenesis of BMMSCs. BMMSCs were incubated for 3 days of adipogenesis in the presence of different concentrations of TUG-891 [0 (Con), 0.1, 0.5, 1, and 5 μM]. (**A**) Effect of low concentrations of TUG-891 on oil red staining (scale bar = 40 μm) and (**B**) quantification of lipid numbers of adipogenic differentiation in BMMSCs; the lipid quantification of each group was carried out using the method described in the Materials and Methods section. (**C**) Effect of low concentrations of TUG-891 on the adipogenic mRNA expression of *Pparγ* and *Fabp4*. (**D**) Western blot analysis of Erk1/2, pErk1/2, Ras, Ras-GRF, p38 and pp38. The expression of each target gene was calculated as the relative expression to β-actin and is represented as normalized fold expression. Data are represented as the mean ± SD of 3 independent experiments. *P < 0.05 and **P < 0.01 compared with the control group.

To figure out why low concentrations of TUG-891 weaken the osteogenesis of BMMSCs, cells underwent adipocyte differentiation in adipogenic medium with different concentrations [0 (Con), 0.1, 0.5, 1, 5 μM] of TUG-891 for 3 days. Interestingly, we found that 0.5 μM TUG-891 significantly increased the ability of adipogenesis, while 5 μM TUG-891 markedly reduced the adipogenic potential compared with controls observed by oil red staining and lipid quantification (Fig. 5A,B, P < 0.05). RT-PCR showed that low concentrations of TUG-891, particularly 0.5 μM, significantly increased the expression level of *Ppar-γ* and *Fabp4* (Fig. 5C, P < 0.05), indicating that low concentrations of TUG-891 played an essential role in adipogenic differentiation rather than the osteogenic differentiation of BMMSCs and, most importantly, that GPR120 participated in bone metabolism in a ligand dosage-dependent manner.

To clarify the underlying mechanism of the adipogenic-inducing effect of low concentrations of TUG-891, the Ras-ERK1/2 pathway was evaluated by western blotting. Consistent with the findings regarding the osteoblast differentiation of BMMSCs, the results showed that low concentrations of TUG-891, particularly 0.1 and 0.5 μM, markedly down-regulated the expression level of pERK1/2 (Fig. 5D). Next, we further tested whether Ras and Ras-GRF could be modulated by low concentrations of TUG-891 under adipogenic conditions. However, the results showed no difference between each group, indicating that a low dose of TUG-891 might inactivate ERK1/2 through other mechanisms (Fig. 5D). We then tested the expression of phosphorylated p38, and the results showed that low doses of TUG-891, particularly 0.1 and 0.5 μM, up-regulated the expression of pp38, which might consequently inactivate ERK phosphorylation to induce the adipogenesis of BMMSCs (Fig. 5D).

### GPR120 activates different integrin subunits to modulate osteogenesis or adipogenesis in a ligand dose-dependent manner

**Figure 6:**
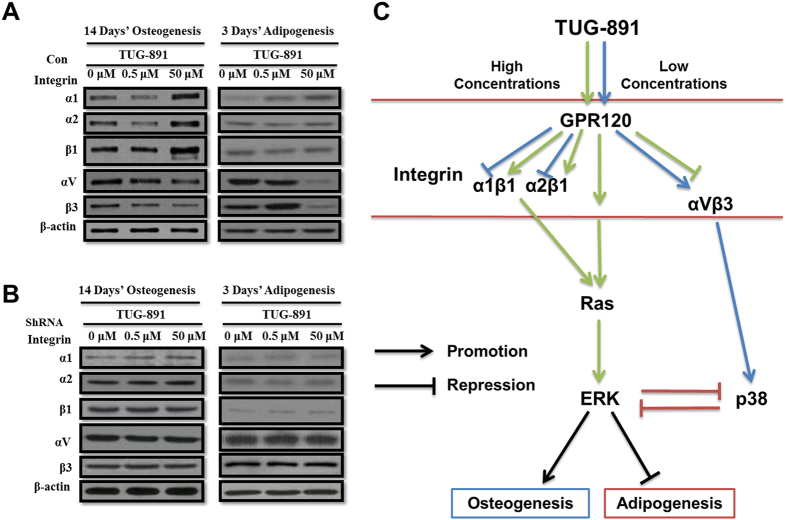
GPR120 activates different integrin family members to modulate osteogenesis or adipogenesis in a ligand dose-dependent manner. BMMSCs were incubated for 14 days of osteogenesis or 3 days of adipogenesis in the presence of different concentrations of TUG-891 (0, 0.5, 50 μM). (**A**) Western-blot analysis of integrin α1, α2, αV, β1 and β3 of control BMMSCs. (**B**) Western-blot analysis of integrin α1, α2, αV, β1 and β3 of GPR120 knocked-down BMMSCs. (**C**) Molecular mode of GPR120 targeting the osteogenesis and adipogenesis of BMMSCs. Arrows indicate to activate the target. The repression symbol indicates the inhibition of the target. The green and blue arrows indicate the pathway of the high concentration and low concentration of TUG-891, respectively, targeting GPR120 and their downstream targets.

To further determine why GPR120 could be the bi-potential mediator to modulate either the osteogenesis or adipogenesis of BMMSCs and how GPR120 could activate ERK and p38 in a ligand dose-dependent manner, the expression of integrin family members was tested by western blotting. In BMMSCs that underwent 14 days of osteogenesis or 3 days of adipogenesis, we found that a high concentration of TUG-891 (50 μM) up-regulated integrin α1, α2, and β1 but down-regulated integrin αV and β3 compared with the control group (0 μM TUG-891). By contrast, a low concentration of TUG-891 (0.5 μM) down-regulated integrin α1, α2, and β1 but up-regulated integrin αV and β3 (Fig. 6A).

To exclude the possibility that TUG-891 directly activated integrin family members, but not through GPR120, we used GPR120-knocked down BMMSCs and subjected cells to 14 days of osteogenesis and 3 days of adipogenesis with a low and high concentration of TUG891, and then, integrin expression was tested by western blotting. Figure 6B showed that neither a high concentration nor a low concentration of TUG-891 affected the expression of integrin family members in GPR120-knocked down BMMSCs, indicating that the activation of different integrin receptors by dose-dependent TUG-891 occurred through GPR120.

Olsen and Volloch indicated in their study that ERK could be activated by integrin α1β1 and α2β1 to promote osteogenesis, while p38 could be activated by integrin αVβ3 to repress ERK and induce adipogenesis^31^. The molecular mechanisms of high and low concentrations of TUG-891 targeting both the osteogenesis and adipogenesis of BMMSCs through GPR120 are depicted in schematic diagram (Fig. 6C).

### GPR120 improves the bone mass and bone structure of ovariectomized mice

**Figure 7:**
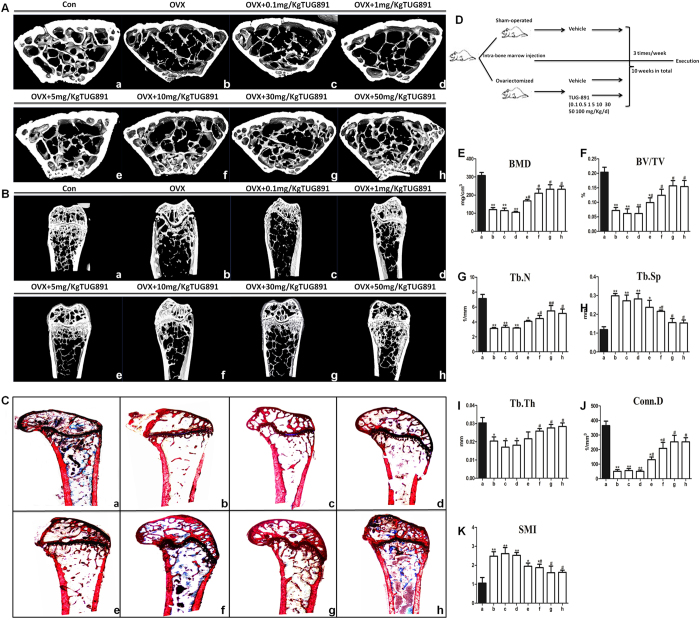
GPR120 improves the bone mass and bone structure of ovariectomized mice. Groups: a: Sham; b: OVX; c: OVX + TUG-891 (0.1 mg/kg); d: OVX + TUG-891 (1 mg/kg); e: OVX + TUG-891 (5 mg/kg); f: OVX + TUG-891 (10 mg/kg); g: OVX + TUG-891 (30 mg/kg); h: OVX + TUG-891 (50 mg/kg). (**A**,**B**) Analysis of micro-CT in the distal metaphyseal femur region. (**C**) Van Gieson (VG) staining of the distal femur. The figure was 40 × and 100 × of the original section. (**D**) Procedure for the *in vivo* injection of TUG-891 among the different groups. (**E**,**K**) Analysis of micro-CT quantification in the distal metaphyseal femur region. The following 3D indices in the defined region of interest (ROI) were analysed: BMD, Conn.D, SMI, Tb.N, Tb.Th, Tb.Sp and BV/TV. *P < 0.05 and **P < 0.01 compared with the sham group. ^#^P < 0.05 and ^##^P < 0.01 compared with the OVX group.

**Figure 8:**
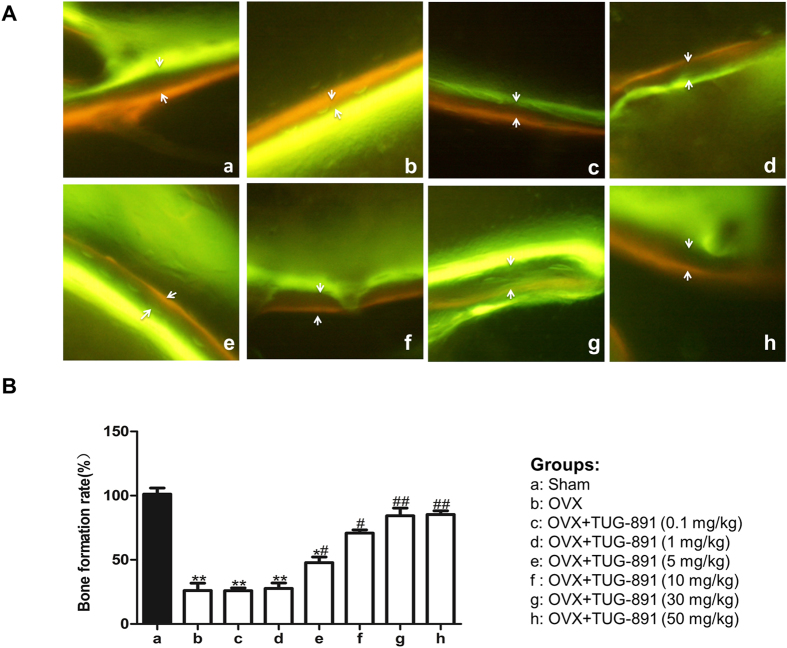
GPR120 increases the bone formation rate (BFR) of ovariectomized mice. Fluorochrome double labelling was performed by intramuscular injection of tetracycline hydrochloride and calcein administered 12 and 2 days before sacrifice. In all of the groups, the fresh osteoids were labelled with tetracycline hydrochloride and calcein. (**A**) Fluorochrome double labelling of each group. (**B**) Quantification of the bone formation rate between each group. Groups: a: Sham; b: OVX; c: OVX + TUG-891 (0.1 mg/kg); d: OVX + TUG-891 (1 mg/kg); e: OVX + TUG-891 (5 mg/kg); f: OVX + TUG-891 (10 mg/kg); g: OVX + TUG-891 (30 mg/kg); h: OVX + TUG-891 (50 mg/kg). *P < 0.05 and **P < 0.01 compared with the sham group. ^#^P < 0.05 and ^##^P < 0.01 compared with the OVX group.

To further evaluate the effect of GPR120 on bone mass and micro-architecture *in vivo*, different concentrations of TUG-891 and vehicle were injected into the proximal femur of ovariectomized (OVX) mice. To avoid the indirect impact of hypodermic injection on bone mass and density, a local direct injection to the bone marrow cavity was adopted in this study. The detailed time course of the *in vivo* administration of TUG-891 is shown in Fig. 7D. When all of the mice were collected at 10 weeks after operation, there were no significant differences in the body weights among all of the groups. The analyses of the trabecular bone of the distal femur showed that ovariectomy reduced the bone mass and deteriorated bone micro-architecture (Fig. 7A,B), as indicated by the decrease in BMD, Conn.D, Tb.N, Tb.Th and BV/TV in OVX mice (Fig. 7E–K, P < 0.05). However, SMI and Tb.Sp were increased (P < 0.05). The treatment of ovariectomized mice with 10, 30 and 50 μmol/kg of TUG-891 in part rescued these bone parameters and improved the micro-architecture of the trabecular bone in the distal femur (Fig. 7A,B). Moreover, we evaluated the changes in bone micro-architecture by VG staining. As shown in Fig. 7C, compared with the sham-operated group, the number of trabeculae decreased and the trabecular space became broader in the OVX group. High concentrations of TUG-891 (10, 30, and 50 μmol/kg) reversed these changes by an elevated number of trabecular bone and a reduction of trabecular bone space, while there were no changes in the concentration groups of 0.1 and 1 μmol/kg. To determine whether the activation of GPR120 could influence the bone formation rate (BFR) of bone *in vivo*, fluorochrome double labelling was performed by intramuscular injection of tetracycline hydrochloride and calcein administered 12 and 2 days before sacrifice. In all groups, the fresh osteoids were labelled with tetracycline hydrochloride and calcein (Fig. 8A). As shown in Fig. 8B, the OVX group demonstrated a significant decrease (P < 0.05) in BFR in contrast to the sham-operated group. However, the BFR of the groups treated with high concentrations of TUG-891 (10, 30, 50 μmol/kg) exhibited a significant increase compared with the OVX group (P < 0.05). Our research demonstrated that high concentrations of TUG-891 could protect the trabecular bone mass via increasing BFR.

## Discussion

The link between bone and fat has been studied for many years, and the role of FFAs, as important lipids in lipid metabolism, in bone metabolic diseases, such as osteoporosis, has been attracting much attention^32^. Understanding the role and underlying mechanism of FFA in affecting skeletal cells will undoubtedly help us to further understand the relationship between fat and bone and explore more efficient anti-osteoporosis drugs.

The best way to prevent osteoporosis in the aging population is to build strong bones in early life through consuming a well-balanced diet (e.g., calcium and ω-6 and ω-3 fatty acids) and following a routine exercise program^33^. Although direct evidence for the beneficial effect of dietary ω-3 fatty acids on human osteoporosis is still lacking, experiments using animal and cell culture models have indicated promising applications of ω-3 PUFA in this disease^20^. Hogstrom *et al*. also reported that the serum phospholipid levels of ω-3 PUFA and DHA were positively related to bone mass density (BMD)^22^. Moreover, there was an available study demonstrating that the tissue levels of ω-3 PUFA were positively correlated with bone mass in postmenopausal women^34^. However, another unsaturated fatty-acid, ω-6 PUFA, was reported to play a negative role in bone formation^35^,^36^,^37^,^38^. Both ω-3 PUFA and ω-6 PUFA exerted their function through GPR120. However, it is unknown why GPR120 was able to mediate a different biological process with a different ligand.

GPR120, as a G protein-coupled receptor, has been reported to accelerate adipocyte differentiation and modulate the secretion of insulin and glycometabolism^16^. Whether GPR120 mediates the osteogenesis-induction effect *in vivo* and *in vitro* remains unclear. In this study, we found that the expression level of GPR120 increased with the induction of osteogenesis in BMMSCs. Next, we knocked down GPR120 in BMMSCs, which showed markedly reduced osteogenesis. Moreover, this effect was modulated by the Ras-ERK1/2 cascade, a finding that was consistent with the results from Katsuma *et al*. that the extracellular signal-regulated kinase (ERK) pathway worked downstream of GPR120 to modulate biologic functions^39^.

The effect of GPR120 on bone metabolism occurred in a ligand dose-dependent manner. High concentrations of TUG-891 significantly promoted osteogenesis via the Ras-ERK1/2 cascade, while low concentrations increased the adipogenesis of BMMSCs by elevating p38 *in vitro*. Our finding of a dosage-dependent effect of the agonist of GPR120 in this study might better explain the different roles of GPR120 in both osteogenesis and adipogenesis in the presence of different ligands. Such situations exist in the physiological process *in vivo*. Along with maturation and aging, the levels of ω-3 PUFA and other “osteo-inductive” FFAs in the body were gradually reduced and the relatively “adipo-inductive” FFAs were elevated. The minute difference among ligand-dependent GPR120 functions and/or the slight affinity difference of the ligands might modulate the biological process and cell fate of BMMSCs. Our findings, for the first time, elucidated the molecular mechanism of osteoblast and adipocyte differentiation mediated by different ligands acting on the same receptor, and GPR120 was demonstrated to be the key molecule between the osteo- and adipo-differentiation of BMMSCs.

However, the cytoplasmic switch downstream of ligand dose-dependent GPR120 in cell fate determination remains unclear. Integrin family members are the principal mediators of the molecular dialogue between cells and their extracellular matrix (ECM) environment^40^,^41^,^42^. Previous studies revealed that αV, α5β1, αvβ3, and β3/β5 integrins are involved in the interaction between osteoblasts and the ECM and affect osteoblast function and bone remodelling^43^,^44^,^45^. Moreover, many studies have indicated that the engagement of α1β1 and α2β1 binding sites markedly activates ERK and activates p38 kinase rather weakly^46^,^47^. The more α1β1 and α2β1 sites that are engaged, the higher are the levels of activated ERK, while the engagement of αvβ3 binding sites strongly activates p38 kinase and rather weakly activates ERK^48^,^49^,^50^,^51^. Olsen and Volloch indicated that α1β1 and α2β1 could activate Ras-Erk to induce osteogenesis, while integrin αVβ3 could activate p38 and subsequently repress the phosphorylation of ERK to promote adipogenesis^31^. Our team also reported that hBMSCs obtained from senile osteoporotic patients gradually lose their osteogenic capability through the abnormal regulation of the integrin α2-ERK-Runx2 signalling pathway. In this study, we showed that high concentrations of TUG-891 from 30 to 100 μM markedly induced the osteogenesis of BMMSCs through the activation of GPR120-integrin α1β1 via the α2β1-Ras-ERK1/2 signalling pathway. Moreover, low concentrations of TUG-891, from 0.1 to 1 μM, promote the adipogenesis of BMMSCs by activating the GPR120-integrin αVβ3-P38-ERK1/2 signalling pathway. These findings indicate that GPR120, the G-protein-coupled fatty acid receptor expressed in BMMSCs, could have a bi-potential effect in modulating the osteogenesis and adipogenesis of BMMSCs in a ligand dose-dependent manner and help us understand more about the relationship between fatty acid and bone metabolism.

Furthermore, the *in vivo* administration of high doses of TUG-891 rescued oestrogen-deficient bone loss, supporting the osteo-inductive effect of GPR120 on BMMSCs. However, there was no obvious effect of low doses. The cause may be two-fold. First, the concentrations of TUG-891 (0.1 and 1 μmol/kg) were too low to markedly impact the bone micro architecture and bone mass *in vivo*. Second, the process and mechanisms of bone remodelling *in vivo* were too complicated, and there might be some paracrine factors that also regulate BMMSCs. Thus, TUG-891 alone at low concentrations did not exhibit a significant effect on bone metabolism. One thing need to mention is that although TUG-891 was showed to be a selective and potent agonist of GPR120, it still has a chance to activate GPR40, and also other receptors/pathways which we have no idea yet. Therefore, future work will keep focusing on the underling effect of TUG-891 and its potent mechanism in bone-related cells such as BMMSCs, osteoblast and osteoclast.

In conclusion, using both *in vivo* and *in vitro* data, we demonstrated that high concentrations of the selective GPR120 agonist TUG-891 played a positive role in protecting against bone loss in ovariectomized mice and improved osteogenesis via the GPR120-integrin α1β1, α2β1-Ras-ERK1/2 signalling pathway. Low concentrations of TUG-891 induced the adipogenesis of BMMSCs *in vitro* via GPR120- integrin αVβ3-p38 and the subsequent inhibition of ERK1/2. Our findings established another link between fat and bone and elucidated the role and function of FFAs in bone metabolism through the receptor GPR120. Above all, as we showed in this study, GPR120 mediates bi-potential differentiation potency of BMMSCs by ligand dosage-dependent manner, which indicates a potential clinical targets and applications. The activation by high dosages of TUG-891 exerts osteogenic effect of BMMSCs, which can be further researched for the treatment of skeletal diseases. Low dosages of TUG-891 markedly induced adipogenesis of BMMSCs, which indicates that GPR120 is metabolically protective, because adipogenesis in human species protects against insulin resistance and diabetes in the context of obesity. In this regard both sides of the bimodal effect of GPR120 may have a protective effect in the context of obesity and will undoubtedly help us to better understand skeletal diseases, such as osteoporosis over the next few decades.

Until now, GPR120 has been suggested to be a potential candidate and target to treat type 2 diabetes and obesity-induced inflammatory disease^52^,^53^. In the prevalence of age- and metabolic-related disorders, our findings highlighted the essential role of GPR120 in mediating the bi-potential differentiation of BMMSCs and the ability of a GPR120 agonist to protect against bone loss *in vivo*. These findings inspire us to consider that GPR120 and its agonist may be a new molecular target and remedy, respectively, to combat osteoporosis for the foreseeable future.

## Materials and Methods

### Animals

Healthy female C57BL/6 mice, weighing 20.7 ± 1.20 g, were obtained from the Experimental Animal Center, Fourth Military Medical University Xi’an, China, and were acclimated to laboratory conditions for 1 week before commencement of the experiment.

For the OVX experiments, 12-week-old mice were bilaterally ovariectomized. The ovaries of sham-operated mice were left intact. The mice were randomly divided into distinct groups: (n = 9 per group; sham-operated/control vehicle; OVX/control vehicle; OVX/TUG-891). Injection of the bone marrow cavity was started after a 48-h recovery period. Three times per week mice received 5 μL of both DMSO as the control vehicle or TUG-891 at a concentration of 0.1, 1, 5, 10, 30 or 50 μmol/kg for the different groups for 10 weeks. The left femurs of the mice were collected, and the adherent tissue was discarded.

### Ethics Statement

All experimental procedures in animals were approved by the Ethics in Animal Research Committee of the Fourth Military Medical University (permission code 20110405-5). All methods were carried out in “accordance” with the approved guidelines.

### Cell culture

BMMSCs were isolated from C57BL/6 mice as previously described^54^, and cells were characterized using mesenchymal stem cell minimal criteria^55^. Briefly, the bone marrow was flushed out of the long bones of mice and plated as a cell suspension (0.5 × 10^6^ cells cm^−2^) in α-MEM supplemented with 15% FBS, 2 mM glutamine, 100 U/ml penicillin, and 100 μg/ml streptomycin. Culture dishes were incubated at 37 °C in a humidified atmosphere of 95% air and 5% CO_2_. The culture medium was replaced every other day. Homogeneous BMMSCs were obtained for three generations. For osteogenic induction, BMMSCs were cultured at 1 × 10^5^ cells/cm^2^ in DMEM expansion medium supplemented with osteogenic supplements, 1 μM dexamethasone, 10 mM β-glycerophosphate, and 50 μg/ml ascorbic acid in the presence or absence of TUG-891 from a concentration of 0.1 to 100 μM for 14 days. For adipogenic induction, cells were cultured at 1 × 10^5^ cells/cm^2^ in an adipogenic hormonal cocktail expansion medium supplemented with 10 μM dexamethasone, 5 μg/ml insulin, and 0.5 mM 3-isobutyl-1-methylxantine in the presence or absence of TUG-891 from a concentration of 0.1 to 5 μM for 3 days. The medium was replaced every other day.

### Transfection of Lentiviral Vectors with shRNA for GPR120

The pGMLV-GFP-vshRNA-GPR120 shRNAi vector was constructed (pGMLV; Shanghai Genomeditech Co. Ltd). In the present study, we constructed 3 vshRNA-GPR120 lentiviral vectors (pGMLV-GFP-shRNA-GPR120) to silence the expression of GPR120 in murine BMMSCs (BMMSCs-vshRNA-GPR120). The three shRNA-targeting sequences for GPR120 were as follows: #1, 5′-GCACCCACTTCCCTTTCTTCT-3′; #2, 5′-GCTCTTCTACGTGATGACAAT-3′; and #3, 5′-GGACCAGGAAATTCCGATTTG-3′. Stably transfected clones were characterized by reverse transcription-polymerase chain reaction (RT-PCR), western blotting and immunostaining.

### Alkaline phosphatase (ALP) staining and activity assay

Cells were washed twice with phosphate-buffered saline (PBS), fixed with 10% formalin in PBS for 30 s, rinsed with deionized water, and stained with a BCIP/NBT alkaline phosphatase colour development kit under protection from direct light. To measure ALP activity, the cells were lysed with RIPA buffer. The samples were then centrifuged at 10,000 × *g* for 5 min, and ALP activity was measured in clear supernatants using an ALP activity assay kit. The total protein concentrations were determined by the Bradford protein assay. ALP activity was normalized to the total protein concentrations.

### Alizarin red staining for the mineralized matrix

Cells were fixed in ice-cold 10% formalin for 20 min and stained with 40 mM alizarin red S (pH 4.4, Sigma Chemical) for 45 min at room temperature. To estimate matrix calcification, the stain was solubilized with 10% cetylpyridinum chloride by shaking for 15 min. The absorbance of the released alizarin red S was measured at 562 nm^56^.

### Oil red O staining and quantification of adipocytes

Adipocyte fat droplets were observed by the oil red O staining method with minor modifications^57^. Cell monolayers were fixed in 4% paraformaldehyde, washed with water, and stained with 0.6% (w/v) oil red O containing 60% isopropanol for 15 min at room temperature. For quantification of adipocytes, cell monolayers were washed extensively with water to remove unbound dye, and 1 ml of isopropyl alcohol was added to the culture dish. Adipocytes were quantified by counting the red pixels in five random microscopic images per well using Adobe Photoshop software. The values were expressed as a percentage of the total pixels in each microscopic image^58^.

### RNA purification and quantitative real-time polymerase chain reaction (qRT-PCR)

Total RNA was purified from cells using Trizol^®^ (Invitrogen). RT-PCR was performed, and the results were analysed as previously described^59^. All of the RT-PCR experiments were performed in triplicate, and the primer sequences were as follows: *Alp* (Fwd) GAGATGGTATGGGCGTCTC, (Rvs) GTTGGTGTTGTACGTCTTGGA; *Runx2* (Fwd) GCACAAACATGGCCAGATTCA, (Rvs) AAGCCATGGTGCCCGTTAG; *Ocn* (Fwd) GACAAGTCCCACACAGCAACT, (Rvs) GGACATGAAGGCTTTGTCAGA; *Fabp4* (Fwd) TGGGAACCTGGAAGCTTGTCTC, (Rvs) GAATTCCACGCCCAGTTTGA; *PPARG* (Fwd) GGAGCCTAAGTTTGAGTTTGCTGTG, (Rvs) TGCAGCAGGTTGTCTTGGATG; *β-actin* (Fwd) CTGGCACCACACCTTCTACA, (Rvs) GGTACGACCAGAGGCATACA.

### Western blotting

Cells were lysed in lysis buffer (50 mM Tris–HCl, pH 7.4, containing 150 mM NaCl, 1% Nonidet™ P-40, 0.1% SDS, 10 mg/ml leupeptin, 10 mg/ml pepstatin A, and 10 mg/ml aprotinin) on ice for 30 min. For western blot analysis, 10 μg of total protein was resolved by 10% SDS-PAGE, and the proteins were transferred onto a PVDF membrane. Anti-GPR120 (1:1000), anti-ERK1/2 (1:5000), anti-p-ERK1/2 (1:2000), anti-Akt (1:2000), anti-PI3K (1:5000), anti-JNK (1:2000), anti-p38 (1:2000), anti-pp38 (1:2000), anti-integrin α1 (1:2000), anti-integrin α2 (1:2000), anti-integrin αV (1:2000), anti-integrin β1 (1:2000), anti-integrin β3 (1:2000), anti-Ras (1:1000), and anti-Ras-GRF1 (1:1000) antibodies were used for immunoblotting. α-Tubulin was used as a loading control. The horseradish peroxidase-conjugated secondary antibody was used at a 1:5000 dilution. Images were analysed using Scion Image software.

### Immunostaining

Cells were fixed in 4% paraformaldehyde for 15 min, permeabilized with methanol for 10 min, and incubated with primary antibodies anti-GPR120 (1:100) antibodies overnight. On the following day, the cells were incubated with a DyLight^®^ 594 secondary antibody (Abcam) for 1 h. After staining nuclei with DAPI (0.5 μg/ml) for 5 min, the cells were analysed under a FV1000 model confocal microscope (Olympus, Tokyo, Japan).

### Assessment of bone micro-architecture and bone mass by micro-computed tomography

The distal femurs were scanned using explore Locus SP Pre-Clinical Specimen micro-CT (GE Healthcare, USA) with an 8-mm resolution, a 50-kV tube voltage and a 0.1-mA tube current. The reconstruction and 3D quantitative analyses were determined using software provided by a desktop micro-CT system (GE Healthcare, USA). Similar settings for scans and analyses were used for all of the samples. In the femur, the scanning regions were confined to the distal metaphysis, extending proximally 2.0 mm from the proximal tip of the primary spongiosa. The trabecular bone region from the vertebral body was outlined for each micro-CT slice, excluding both the cranial and caudal endplate regions. The following 3D indices in the defined region of interest (ROI) were analysed: bone mineral density (BMD), connectivity density (Conn.D), structure model index (SMI), trabecular number (Tb.N), trabecular thickness (Tb.Th), trabecular separation (Tb.Sp) and relative bone volume over the total volume (BV/TV, %). The operator who conducted the scan analysis was blinded to the procedure associated with the specimens.

### Histological examination by Van Gieson (VG) staining

The left femur of all of the mice were collected and fixed in 4% paraformaldehyde for 48 h. After dehydration and embedding, the distal femur was embedded in polymethyl-methacrylate (PMMA) and processed into 240-mm-thick sections in the coronal plane using a rotation microtome. Subsequently, all of the sections were hand-grounded to a thickness of 20 mm for VG staining, which was used for staining collagen fibre^59^.

### Fluorochrome double labeling

Fluorochrome labelling of the bones was performed by intramuscular injection of tetracycline hydrochloride (20 mg/kg) and calcein (5 mg/kg) administered 12 and 2 days before sacrifice, respectively. Next, the left femurs were fixed, dehydrated, embedded and sliced (10 μm). At the point of intersection, the distance between the middle of the two fluorescein labels was measured by a magnification of ×200. The BFR was then calculated by dividing the distance between the two labels by the inter-labelling period in days.

All methods were carried out in “accordance” with the approved guidelines.

### Statistical analysis

Data were expressed as means ± S.D. of multiple repeats of the same experiment (n = 3). The data for these measurements were analyzed by one-way analysis of variance (ANOVA) with subsequent post hoc multiple comparisons by Dunnett’s test. Statistically significant values were defined as P < 0.05.

## Additional Information

**How to cite this article**: Gao, B. *et al*. GPR120: A bi-potential mediator to modulate the osteogenic and adipogenic differentiation of BMMSCs. *Sci. Rep*. **5**, 14080; doi: 10.1038/srep14080 (2015).
