# Author Correction: GPR120: A bi-potential mediator to modulate the osteogenic and adipogenic differentiation of BMMSCs

**DOI:** 10.1038/s41598-020-73528-9

**Published:** 2020-10-08

**Authors:** Bo Gao, Qiang Huang, Qiang Jie, Wei-Guang Lu, Long Wang, Xiao-Jie Li, Zhen Sun, Ya-Qian Hu, Li Chen, Bao-Hua Liu, Jian Liu, Liu Yang, Zhuo-Jing Luo

**Affiliations:** 1 Institute of Orthopedic Surgery, Xijing Hospital, Fourth Military Medical University, Xi’an, 710032 People’s Republic of China; 2 Lanzhou General Hospital of Lanzhou Military Command, Lanzhou, 730050 Gansu People’s Republic of China; 3 KMEB, Molecular Endocrinology, Campusvej 55, 5230 Odense M, Denmark; 4 Health Science Center, Shenzhen University, 3688 Nanhai Ave, Shenzhen, 518060 People’s Republic of China

Correction to: *Scientific Reports* 10.1038/srep14080, published online 14 September 2015

This Article contains errors.

In Figure 2A, the images for the “ShRNA” and “ShRNA + TUG 50 µM” conditions are incorrect. The correct Figure 2 appears below, as Figure 1.

**Figure 1:**
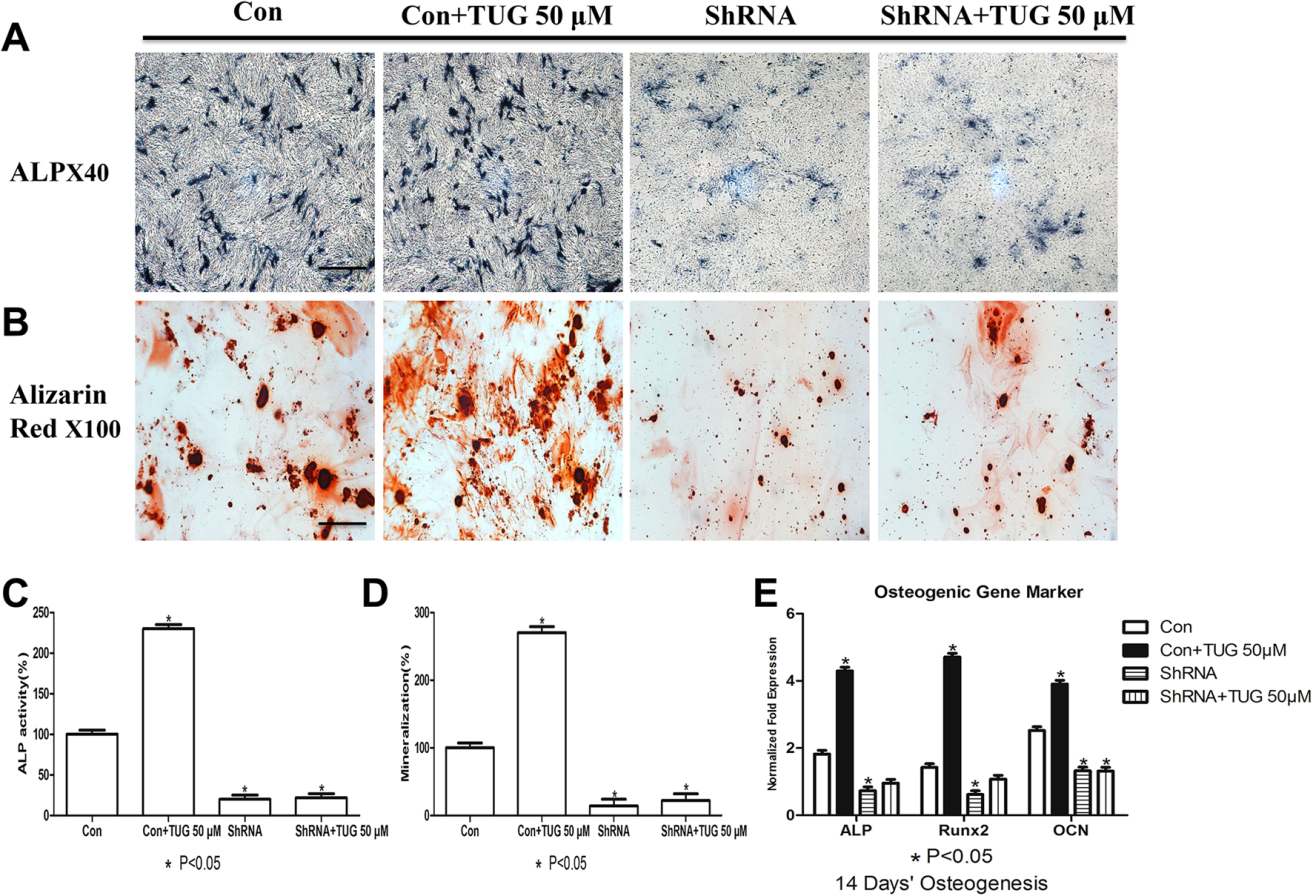
A corrected version of Figure 2 in the Article.

**Figure 2:**
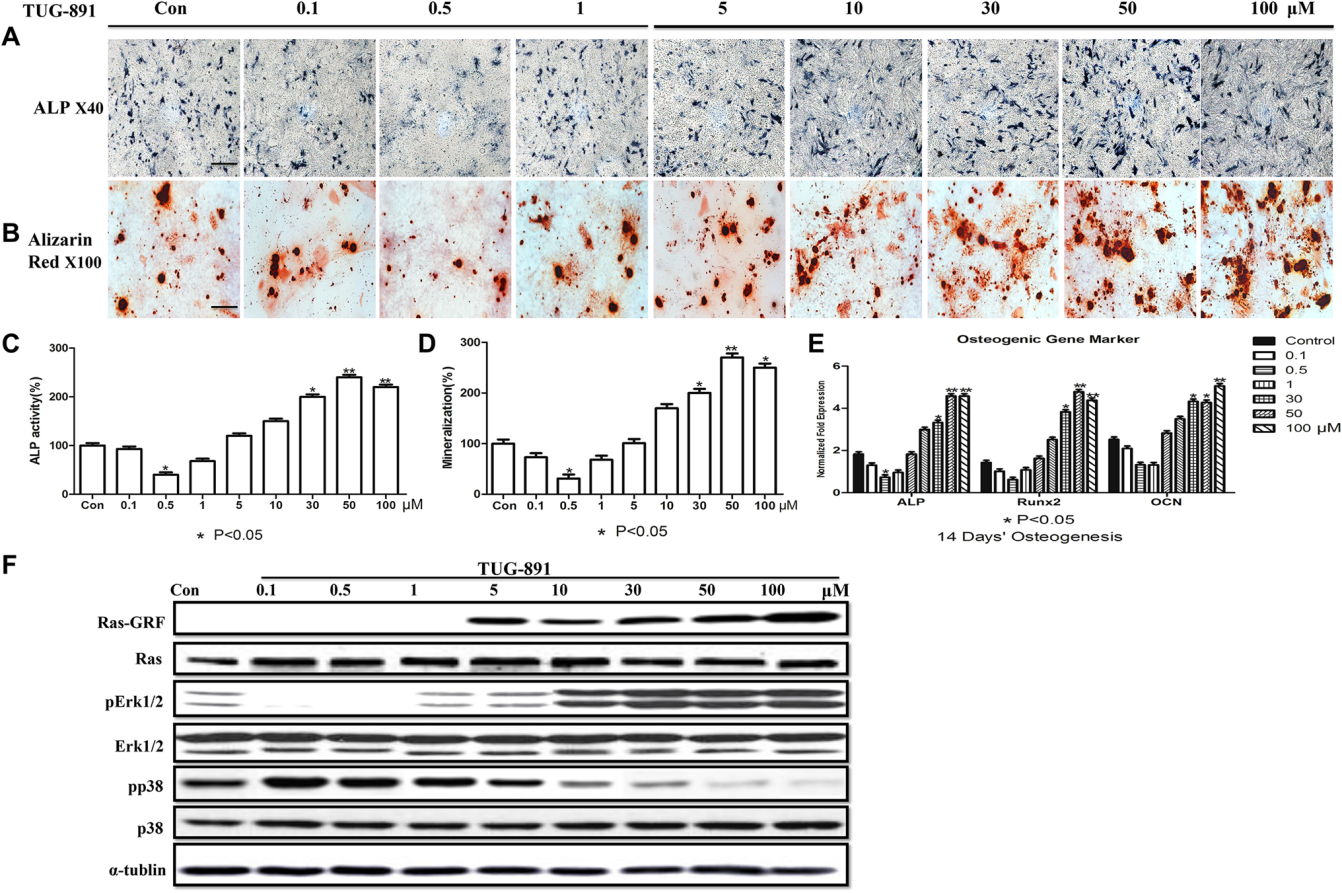
A corrected version of Figure 4 in the Article.

In Figure 4A, the images for 0.5 µM, 5 µM, 30 µM, and 50 µM treatments are incorrect. The correct Figure 4 appears below, as Figure 2.

